# Supplementary material for: Medical teleconsultation from the patient’s perspective. A demographic segmentation
Source: Eur J Health Econ. 2025 Jan 30;26(6):1023–39. doi: 10.1007/s10198-024-01753-4 (PMC12310798; doi:10.1007/s10198-024-01753-4)
Supplement: Supplementary file 1 — Supplementary file1 (PDF 1420 KB) [file 10198_2024_1753_MOESM1_ESM.pdf]

GILES J.M. COLLINGE GENOVESE

PSYCHOLINGUIST

Professor of English/Translator/Proofreader

E: gcg51@hotmail.com /Calle Pilistra 1 -8ª/41008 Seville/ Spain

To whom it may concern:

The paper "**Medical teleconsultation from the patient's perspective: A socio-demographic segmentation**" has been proofread by the abovementioned professional, UK native translator.

28th. October 2024

ENGLISH LANGUAGE SERVICES · GILES COLLINGE · 22 60 10 - SEVILLA
