# Supplementary material for: Medical teleconsultation from the patient’s perspective. A demographic segmentation
Source: Eur J Health Econ. 2025 Jan 30;26(6):1023–39. doi: 10.1007/s10198-024-01753-4 (PMC12310798; doi:10.1007/s10198-024-01753-4)
Supplement: Supplementary file 2 — Supplementary file2 (DOCX 657 KB) [file 10198_2024_1753_MOESM2_ESM.docx]

**Medical teleconsultation from the patient’s perspective: A socio-demographic segmentation.**

**Abstract:** Medical teleconsultation is a tool that is here to stay among the services offered by health systems. Therefore, it is important to understand the process of adopting this technology. However, most studies have endorsed the point of view of health professionals. Our research adopts the patient's point of view with a sample of 1500 patients who used teleconsultation in Spain between May and November 2022, therefore in a post-COVID-19 scenario. We started from a technology acceptance model, UTAUT, and applied a novel segmentation technique: Pathmox. As a result, we have obtained six segments of patients using teleconsultation with differentiated technology acceptance processes, and we also propose strategies adapted to each of them.

**Keywords**: Telemedicine, Teleconsultation, Demographic segmentation, Technology acceptation, Pathmox.

**1.Introduction**

One of the priority objectives of the European Union is to ensure that its citizens have access to the health systems of its Member States. EU countries aim to ensure that their health systems provide affordable, equitable and high-quality medical care, emphasising that health care is a fundamental human right (European Commision 2024a). In order to achieve these goals, it is essential to advance the process of digitalisation of health systems (Majcherek et al. 2024). From an economic perspective, the European Commission itself estimates that improved access to, and exchange of, health data could save €5.5 billion over the next 10 years (European Commision 2024b).

Healthcare systems have used advances in information and communication technologies since their inception. For example, the first steps in telemedicine date back to the mid-20th century, with the use of the telephone as a means of medical consultation. Later, in the 1970s, during the space race, NASA developed telemedicine tools to provide health care to its astronauts. However, the recent COVID-19 pandemic was a turning point in the development of these tools (Sageena, Sharma, and Kapur 2021). Today, in a society heavily influenced by technology at all levels, all kinds of devices such as mobile phones, cameras or wearable biosensors have been incorporated to obtain clinical information (Adeghe, Okolo, and Ojeyinka 2024). This technological advance affects individuals and society as a whole, implying a digital transformation (Gradillas and Thomas 2023) in the field of health systems. However, this digital transformation does not affect everyone equally. There are important differences between individuals and social groups when it comes to coping with the use of digital technologies (Bockshecker, Hackstein, and Baumöl 2018; Kraus et al. 2021). In this digital context, the patient plays an increasingly active role (Yang et al. 2022). And among all the digital services available, a key service is the medical consultation as a moment of interaction between doctor and patient. The digital environment has given way to this (Hernández-Zambrano et al. 2021).

The study by (Dahlgren et al. 2024) suggests that direct-to-consumer telemedicine use might lead to increased medical service utilisation in the short and intermediate term. This trend could result from the ease of access and the availability of technology but could also encourage overuse or unnecessary use if a strong connection to primary care is not established. To address these risks, the authors underscore the need to identify which diagnoses and treatments are suitable for direct-to-consumer telemedicine, aiming to maximise its cost-effectiveness while curtailing its use where this could be inappropriate. This requires a thorough examination of behaviour patterns among telemedicine users, which could offer insights to refine public policies and foster efficient practices. Meanwhile, post-COVID-19, Europe is turning to telemedicine to bridge gaps in access to primary healthcare services. A key component of this strategy is the establishment of integrated primary care centres that harness technology to manage electronic medical records and promote collaborative clinical documentation. This model is particularly beneficial for healthcare professionals who practise telemedicine. These centres can substantially enhance coordination among professionals and ensure high-quality care for patients, even in remote or underserved areas (Garattini, Badinella Martini, and Zanetti 2021). To achieve this objective, a thorough understanding of teleconsultation users' practices and preferences is essential, as this information could drive the effective management and optimisation of these centres.

There are a significant number of studies on medical teleconsultation (Garavand et al. 2022), most of them from the point of view of health care professionals. However, there are still few studies from the patients' point of view (Baudier et al. 2022). Moreover, we know from other highly digitised sectors, such as electronic banking (Arenas-Gaitán, Villarejo Ramos, and Peral-Peral 2019) or online social networks (Villarejo-Ramos, Peral-Peral, and Arenas-Gaitán 2019) that the process of acceptance of new technologies is not homogeneous among all users. There is heterogeneity, differences, between segments of users of online services. We believe that medical teleconsultation is no exception.

The aim of this paper is to analyse the acceptance of teleconsultation technology by differentiating between different types of users. In order to achieve this objective, we will translate it into several research questions. The first research question examines the acceptance process of teleconsultation from the patient’s perspective. To address this, we will utilise the Unified Theory of Acceptance and Use of Technology (UTAUT) (Venkatesh et al. 2003) allows for an adequate analysis of the technological acceptance process of medical teleconsultation. This model has been widely used in the field of telemedicine (Garavand et al. 2022), although there are still few studies for the case of medical teleconsultation. Nonetheless, we understand that this acceptance process may be influenced by the characteristics of individuals. Therefore, the second research question is whether the socio-demographic characteristics of the users, such as their age, level of education or income, influence the process of acceptance of teleconsultation. To answer this question, we will use the Pathmox technique (Lamberti, Banet-Aluja, and Sanchez 2017), which allows us to distinguish different groups, in the form of a tree, using distinct segmentation criteria at the same time. Given that each segment has its own characteristics, we will analyse the similarities and differences between the different segments obtained above with respect to their medical teleconsultation acceptance process. To test this, we will use the PLS-MGA multigroup analysis (Sarstedt, Ringle, and Hair 2017a).

The paper is organised in a manner that first provides a comprehensive review of the relevant literature. Subsequently, the methodology employed in the study is explained. The main findings of the analysis are then presented, followed by a discussion of the results in comparison to other studies. The academic, managerial, and social implications of the research are also highlighted. The paper concludes with an assessment of the limitations of the research and suggestions for future research avenues.

**2. Theoretical Framework**

**2.1. Telemedicine & Medical Teleconsultation**

Telemedicine, as defined by the World Health Organisation (WHO), is a healthcare practice that leverages interactive audio-visual and data communications technologies to provide medical services, including diagnosis, treatment, consultation, health education and the exchange of medical data (Albahri et al. 2021). The use of telemedicine has been widely accepted as an effective solution for remote healthcare, particularly in areas with limited or inaccessible healthcare facilities (Nittari et al. 2020a). With the widespread adoption of technology, telemedicine has gained popularity by enabling medical specialists to offer their services to patients without the need for physical travel (Nittari et al. 2020b). Telemedicine represents the progression of healthcare into the digital age and is poised to shape the future of medical practices (Chellaiyan, Nirupama, and Taneja 2019).

Telemedicine, as it is practised today, utilises the computing devices of either the patient or healthcare professional, along with low-cost proprietary equipment such as smartphones, biosensors and laptops, to gather clinical data, thus obviating the need for extensive training (Mazzuca et al. 2022). This has resulted in a reduction of travel expenses and saved time, while also lowering medical costs and facilitating greater access to specialist medical practitioners for the general public without the need to interrupt their daily activities, thereby increasing overall productivity. Additionally, it has alleviated the workload of healthcare professionals by decreasing missed appointments and cancellations, which in turn has increased revenue and patient throughput, and has led to improved follow-up care and overall health outcomes (Banning et al. 2022). The advent of telemedicine has sparked a migration of healthcare from traditional clinics and hospitals to the home environment (López, Lam, and Thota 2021). As can be seen, telemedicine is a broad concept. Therefore, Tables 1a and 1b summarise the main types of telemedicine according to different criteria and their main applications today.

Table 1a: Telemedicine Types

| Category | Telemedicine Type | Description |
| --- | --- | --- |
| According to the timing of the information transmitted | Real time or synchronous telemedicine | Where the sender and receiver are online at the same time and information is transferred live. |
|  | Store‑and‑forward or asynchronous telemedicine | Where the sender stores information in databases and sends it to the receiver to review at their convenience. |
|  | Remote Monitoring type of telemedicine | Uses various technological devices to remotely monitor a patient's health and clinical signs. |
| According to the interaction between the individuals involved | Health professional to health professional | Provides easier access to speciality care, referral, and consultation services. |
|  | Health professional to patient | Delivers healthcare to underserved populations by providing direct access to a medical professional. |
| Own elaboration based on Chellaiyan, Nirupama, and Taneja (2019) | | |

Table 1b: Telemedicine Applications

| Category | Telemedicine Application | Description |
| --- | --- | --- |
| Educational | Tele‑education | Interactive long-distance learning programme for training and updates on recent medical advances. |
|  | Tele‑conferencing | Virtual discussions and interactions between doctors during workshops, conferences, and continuing medical education programmes. |
|  | Tele‑proctoring | Remote mentoring and evaluation of surgical trainees using advanced video conferencing equipment. |
| Healthcare Delivery | School‑based health centres | Manages chronic conditions like asthma, diabetes, and obesity by providing school nurses remote access to specialist medical opinions. |
|  | Correctional facilities | Addresses inmates' healthcare needs without the costs and risks of inmate transportation or requiring specialist visits. |
|  | Mobile health clinics | Provides quick access to remote physicians or medical specialists. |
|  | Shipping and transportation | Helps avoid evacuations and unscheduled diversions during medical emergencies. |
|  | Industrial health | Offers on-site medical management and triage advice. |
| Healthcare Management | Tele‑health care | Uses ICT for preventive and promotive healthcare, including teleconsultation and tele follow-up. |
|  | Tele‑home health care | Monitors patients remotely with a Computer Telephone Integrated (CTI) system for 24-hour vital signs monitoring. |
|  | Specialties | Includes tele-ophthalmology, tele-psychiatry, tele-cardiology, tele-surgery, etc. |
|  | Diagnostic services | Provides tele-radiology and tele-endoscopy services. |
| Emergency and Preventive Care | Disaster management | Ideal for disaster-stricken regions with disrupted connectivity, using satellite and customised telemedicine software. |
|  | Screening of diseases | Uses telemedicine technologies for early detection and screening of diseases. |

Own elaboration based on Chellaiyan, Nirupama, and Taneja (2019)

The implementation of telemedicine in healthcare has faced numerous challenges, hindering its widespread adoption. These challenges include a lack of awareness among patients, the high cost of implementation, operational inefficiencies, difficulties in conducting physical examinations, a general perception that virtual care is not as effective as in-person care, financial implications, legal and regulatory hurdles, and concerns about medical liability (Makhni, Riew, and Sumathipala 2020).

In this context, it is crucial to understand medical teleconsultation as a pivotal component within the realm of telemedicine. Advances in technology, such as computers, smartphones and tablets, have elevated the traditional doctor-patient and doctor-doctor communication methods beyond just auditory means (Liu et al. 2019). The ability to transmit laboratory results, diagnostic images, videos, and even conduct video consultations to examine a patient's own pathology, has been demonstrated through numerous studies to be highly effective. Researchers like Augusterfer et al. (2020) and Mishkin et al. (2022) have analysed teleconsultations in the fields of psychology and psychiatry, and despite acknowledging the need for professional training, they highlight the benefits and potential for growth. Similarly, Wright and Honey (2016) studied the application of telemedicine in home care, while Vural and Ramadan (2019) explored its effectiveness in emergency situations. Furthermore, it has been applied to address the consequences of gender-based violence (Vranda and Cicil 2022) and has been utilised in consultations related to the COVID-19 pandemic with high levels of patient satisfaction (Blanco Terés et al. 2022).

Vranda and Cicil (2022) posit that the success of consultation tools is contingent upon a number of factors, including feasibility, device size, enhancement of face-to-face interaction, capability of transmitting patient data, data type, user-friendly interface and portability, and a synchronous or asynchronous mode of operation. The interaction can occur through a variety of means, such as mobile applications, text-based methods using specialised smartphone applications or chat-based systems and platforms (e.g., WhatsApp, Google Hangouts, Facebook Messenger), video chat platforms (e.g., Skype, Facetime), and even asynchronous media like emails and faxes. While each consultation tool has its own unique advantages and disadvantages, it is however evident that the field of medical teleconsultation is undergoing continuous evolution due to technological advancements (Vural and Ramadan 2019b).

From the patient's perspective, medical teleconsultation has been studied through theories of technology adoption, with theoretical frameworks such as the Technology Acceptance Model (TAM) and UTAUT (Baudier et al. 2021; Lu et al. 2024; Rouidi et al. 2022). The TAM was developed to be applied to technologies at the workplace level (Davis 1985; Davis, Bagozzi, and Warshaw 1989). The TAM model is based on the idea that the acceptance of a technology depends on its usefulness and ease of use (Davis 1989). Subsequently, the UTAUT model (Venkatesh et al. 2003) is a revision and extension of the TAM with contributions from other related theories, incorporating concepts such as social influence or facilitating conditions. We believe that UTAUT offers a suitable conceptual framework to study the process of adoption of teleconsultation.

More specifically**,** UTAUT (Venkatesh et al. 2003) proposed four latent variables that determine user acceptance and usage behaviour (USE): performance expectancy (PE), effort expectancy (EE), social influence (SI), and facilitating conditions (FCs). PE is defined as the degree to which a person using an application believes that this will help him or her achieve a better job performance. EE is defined as the degree of perceived ease of use of an application. SI is defined as the degree to which an individual perceives that important others believe that he or she should use an application. FCs are defined as the extent to which an individual believes that an organisational/technical infrastructure exists to assist the use of an application. These four constructs directly affect behavioural intention (BI). In addition, behavioural intention directly influences the use of the technology, and the facilitating conditions directly determining use behaviour of technology. It can be posited that an increase in perceptions of PE, EE, SI and FC will result in an increase in the utilisation of a technology through BI (Venkatesh et al. 2003). Based on this idea, we formulate the following hypothesis:

*H1: The UTAUT model provides a robust theoretical basis for examining the adoption process of medical teleconsultation from the user’s perspective.*

**2.2. Exploring the Intersection of Family Structures, Socio-demographics, and Technology Adoption**

Traditionally, the application of models to analyse a reality has assumed that the data under analysis comes from a homogeneous population. However, in the field of social sciences, and more specifically in the study of human behaviour, this premise is often unrealistic, given that individuals are likely to exhibit heterogeneity in their perceptions and evaluations of a phenomenon (Matthews et al. 2016; Sarstedt 2008; Sarstedt, Ringle, and Hair 2017b). This assertion applies to the use of medical teleconsultation services as well. Family characteristics, such as size and composition, as well as individuals' level of technology literacy, play a crucial role in the adoption of these new technologies (Creaser et al. 2022). Education, training, the number of household members, and the geographic location also influence the uptake and usage of new technologies, and some studies even consider race and income (Porter and Donthu 2006). For instance, families with children or older members tend to exhibit different behaviours compared to families without such members (Kavandi and Jaana 2020; Lindsay 1968; Sharma 2013). In this regard, we present some demographic variables that have been shown to affect the behaviour of technology users:

*Age*

Numerous studies have highlighted age as the most influential demographic factor affecting technology adoption (Bhatnagar and Ghose 2004; Binesh and Baloglu 2023; Liu et al. 2020). This phenomenon often results from the natural decline in cognitive abilities or older adults’ self-perception of feeling aged. The belief that their cognitive skills have diminished becomes a barrier to adopting new technology (Farivar, Abouzahra, and Ghasemaghaei 2020). Additionally, the literature review suggests that seniors’ perception of the complexity of interactive technology significantly impacts their adoption decisions. In this respect, telemedicine applications can be complex.

*Educational level*

Indeed, research consistently highlights the impact of the education level on technology adoption (Bhatnagar and Ghose 2004; Binesh and Baloglu 2023). The theory of learning suggests that individuals with lower education levels may struggle due to simpler cognitive structures, hindering their ability to adapt to new environments (Paas and van Merriënboer 2020). In contrast, those with higher education tend to approach technology with less apprehension (Schönauer and Glanz 2022). Their awareness and positive attitudes towards new technology contribute to higher adoption rates.

*Income*

There is a significant body of research indicating that income significantly affects the process of technology adoption (Bhatnagar and Ghose 2004; Binesh and Baloglu 2023). Lower-income consumers tend to be more cost-sensitive and resist investing in new technology(Schuetz and Venkatesh 2020). Higher income levels are associated with greater self-confidence, leading to a higher perceived ability to use new technology. Lower-income individuals often view new technology as useless. Adopters of new technology generally have higher income levels than non-adopters.

However, the analysis of these variables separately gives a partial view of the reality. There may be connections between these socio-demographic variables at different levels which need to be taken into account. For example, despite the growing interest in understanding the uptake of information technologies in the healthcare sector from the patient's perspective, the majority of studies focus specifically on the elderly demographic. This is demonstrated by Kavandi and Jaana’s (2020) findings which indicate that 51% of research on the adoption of Health Information Technologies (HIT) by older adults fails to consider an adoption model or framework. There are conflicting results regarding the impact of socio-demographic variables, such as age, gender, and education, on HIT adoption in older adults, suggesting that a broader age range should be studied to fully understand these effects (Chua, Chem, and Wong 1999). On the other hand, Chimento-Díaz et al. (2022) found, in their study of technology adoption in those over 64 years old based on the TAM, that factors such as younger age, higher education, and zest for life positively influence acceptance of technology use in this socio-demographic. Lastly, Tsertsidis, Kolkowska, and Hedström (2019) observed that technology acceptance post-implementation is influenced by multiple factors, including age, with views on technology changing for the better as older adults realise its various benefits in their daily lives.

In summary, the current body of literature regarding the acceptance of health technologies, particularly teleconsultation, lacks sufficient exploration from the patient's perspective, especially with regards to a more comprehensive analysis of the influence of socio-demographic factors. Based on the analysis of the above literature, we propose the following hypothesis:

***Hypothesis 2:****The combination of socio-demographic characteristics, including age, education, and income levels, among users of medical teleconsultation allows for the identification of statistically distinct segments based on their technology acceptance behaviors.*

**3. Methodology**

**3.1 Scales of Measurement**

The measurement scales used in our investigation had been rigorously evaluated and validated in prior research. The scales used to assess Performance Expectancy, Effort Expectancy, Facilitating Conditions, Social Influence and Behaviour Intention were adapted from the UTAUT model proposed by Venkatesh et al. (2003) and Venkatesh, Thong, and Xu (2012). Meanwhile, the scale for measuring the utilisation of teleconsultation was derived from (Kwon and Wen 2010). The variables pertaining to the UTAUT model were quantified using five-point Likert scales.

In addition, a comprehensive examination of various socio-demographic factors associated with the participants was carried out, including their age and level of education. Furthermore, inquiries regarding the composition of their household were conducted, including the size of the household, the presence of minors, and the number of individuals over 70 years of age residing within the household. These socio-demographic variables may be either qualitative, such as educational attainment, or numerical, such as age.

To ensure that the results of our analysis are free from Common Method Bias, we have employed the (Kock 2015) test to assess the Variance Inflation Factor (VIF) of our variables. Our findings indicate that all the VIF levels are below 3.3, thereby guaranteeing the absence of Common Method Bias in our study.

**3.2 Sample**

We utilised a non-probability sample of telemedicine users. The data were collected for Spain as a whole between May and November 2022. This period followed the mandatory confinement in Spain due to the Covid-19 pandemic from March to June 2020. To ensure the suitability of participants, we required them to be over 18 years old and to have used medical teleconsultation services within the past year. We engaged a company specialising in electronic questionnaire data collection. In total, we obtained a sample of 1500 individuals. Additionally, we implemented a filtering process to ensure sample quality. Specifically, we excluded questionnaires completed in less than three minutes, considering this duration insufficient for reliable responses. The average completion time for the questionnaire was just over five minutes. Similarly, we removed questionnaires with inconsistent responses. As a result of this process, we obtained a final sample of 1412 individuals for our analyses. A priori, this is a sufficient sample (Soper 2023; Westland 2010) for the proposed structural model, with an anticipated effect size of 0.11 and a desired statistical power level of 0.8. Of the respondents, 64.4% were male and 35.5% were female. The majority of the participants, 68.2%, resided in urban areas with a population of more than 50,000 while 31.8% lived in rural locations. In terms of education, 1.7% had no or basic education, 59.3% had secondary education, and 39% had a university education. Further demographic details on the sample can be found in the accompanying Table 1.

Table 1: Sample demographic characteristics

|  | Min. | Max. | Average |
| --- | --- | --- | --- |
| Age | 18 | 74 | 38.6 |
| Household size | 1 | 10 | 3.6 |
| Number of children under 18 in the household | 0 | 6 | 1.1 |
| Number of people over 70 in the household | 0 | 5 | 0.2 |

The Spanish health system follows the Beveridge model. This model is characterised by tax-based financing, universal access, salaried or capitated doctors, a minor role for the private sector and a strong state involvement in management. In the European context, other states that follow this model include Portugal, Italy, the United Kingdom, Ireland, Denmark, Finland, and Sweden.

**3.3 Statistical Tools**

To conduct our study, we employed several statistical methods. Firstly, we used structural equation modelling, specifically PLS-SEM, to validate our UTAUT model. This model serves as a foundation for the creation of Pathmox (Lamberti et al. 2017), which was utilised to analyse the diversity in the responses, leading to a segmentation of non-face-to-face consultation users. The Pathmox technique relies on constructing a binary tree to detect population segments with diverse behaviours in relation to the proposed structural equation model, PLS. Finally, we employed multi-group analysis, MGA-PLS (Cheah et al. 2020; Latan and Noonan 2017), to assess and examine the behavioural differences between the identified segments.

**4. Results**

**4.1. PLS-SEM**

The Partial Least Squares Structural Equation Modelling (PLS-SEM) methodology encompasses two distinct stages: the examination of measurement scales, followed by the evaluation of the structural model. To present the outcomes of this analysis, in our reporting we shall adhere to the guidelines put forward by Hair et al. (2019) .

At the onset of our analysis of the measurement scales, it is imperative to emphasise that all the constructs were deemed to be reflective in our study. To verify the reliability of the individual items, we evaluated each item's loading value and confirmed that they all exceeded the recommended 0.7. The reliability of the constructs was further established through Composite Reliability and Cronbach's Alpha, both of which revealed values above 0.7, in line with the established literature (Hair et al. 2019). The convergent validity was established through the Analysis of Variance Extraction (AVE), yielding values above the threshold of 0.5. These findings are presented in Table 2. To ensure discriminant validity, we employed the Fornell and Larcker (1981) and HTMT tests (Henseler, Ringle, and Sarstedt 2015), as presented in Table 2 and Table 3. Overall, our results demonstrate the suitability of the measurement scales used in the study.

Table 2: Indicators of measurement scales

| **Effort** **Expectancy (EE)**  AVE:  CR:  CA: | **Global**  0.748  0.922 0.862 | **Nod 4**  0.668  0.889  0.834 | **Nod 5**  0.701  0.903  0.857 | **Nod12**  0.825  0.950  0.929 | **Nod13**  0.850  0.958  0.941 | **Nod14**  0.685  0.896  0.845 | **Nod15**  0.785  0.936  0.909 |
| --- | --- | --- | --- | --- | --- | --- | --- |
| Learning how to use teleconsulting is easy for me. | 0.851 | 0.805 | 0.796 | 0.903 | 0.925 | 0.834 | 0.858 |
| My interaction with teleconsulting is clear and understandable. | 0.891 | 0.858 | 0.865 | 0.919 | 0.933 | 0.909 | 0.911 |
| I find teleconsulting easy to use. | 0.884 | 0.846 | 0.862 | 0.939 | 0.931 | 0.812 | 0.911 |
| It is easy for me to become skilful at using teleconsulting. | 0.832 | 0.755 | 0.822 | 0.871 | 0.898 | 0.747 | 0.863 |
| **Performance Expectancy (PE)**  AVE:  CR:  CA: | 0.830  0.900 0.830 | 0.772  0.910  0.853 | 0.800  0.923  0.875 | 0.852  0.945  0.914 | 0.890  0.960  0.938 | 0.885  0.959  0.935 | 0.851  0.945  0.913 |
| I find teleconsulting useful in my daily life. | 0.915 | 0.891 | 0.908 | 0.929 | 0.936 | 0.932 | 0.927 |
| Using teleconsulting increases my chances of achieving things that are important to me. | 0.903 | 0.843 | 0.902 | 0.901 | 0.942 | 0.934 | 0.921 |
| Using teleconsulting helps me accomplish things more quickly. | 0.916 | 0.900 | 0.873 | 0.940 | 0.952 | 0.957 | 0.920 |
| **Social Influence (SI)**  AVE:  CR:  CA: | 0.883  0.958  0.934 | 0.833  0.937  0.900 | 0.840  0.940  0.905 | 0.950  0.983  0.974 | 0.923  0.973  0.959 | 0.909  0.968  0.950 | 0.923  0.973  0.958 |
| People who are important to me think that I should use teleconsulting | 0.937 | 0.912 | 0.906 | 0.972 | 0.965 | 0.918 | 0.960 |
| People who influence my behaviour think that I should use teleconsulting. | 0.939 | 0.909 | 0.911 | 0.983 | 0.959 | 0.970 | 0.957 |
| People whose opinions I value prefer that I use teleconsulting. | 0.943 | 0.917 | 0.932 | 0.969 | 0.959 | 0.972 | 0.965 |
| **Facilitating Conditions (FC)**  AVE:  CR:  CA: | 0.705  0.905  0.862 | 0.657  0.884  0.827 | 0.632  0.873  0.807 | 0.821  0.948  0.927 | 0.781  0.934  0.906 | 0.617  0.866  0.808 | 0.711  0.908  0.870 |
| I have the resources necessary to use teleconsulting. | 0.853 | 0.854 | 0.770 | 0.922 | 0.894 | 0.771 | 0.834 |
| I have the knowledge necessary to use teleconsulting. | 0.857 | 0.835 | 0.816 | 0.917 | 0.912 | 0.779 | 0.833 |
| Teleconsulting is compatible with other technologies I use. | 0.839 | 0.763 | 0.805 | 0.903 | 0.902 | 0.827 | 0.863 |
| I can get help from others when I have difficulties with teleconsulting. | 0.810 | 0.788 | 0.790 | 0.881 | 0.824 | 0.764 | 0.843 |
| **Behavioural Intention (BI)**  AVE:  CR:  CA: | 0.812  0.928  0.884 | 0.762  0.906  0.844 | 0.790  0.918  0.867 | 0.816  0.930  0.887 | 0.853  0.946  0.914 | 0.861  0.949  0.919 | 0.839  0.940  0.904 |
| I intend to continue using teleconsulting in the future. | 0.880 | 0.856 | 0.865 | 0.869 | 0.898 | 0.916 | 0.898 |
| I will always try to use teleconsulting in my daily life. | 0.909 | 0.877 | 0.901 | 0.907 | 0.934 | 0.927 | 0.927 |
| I plan to continue to use teleconsulting frequently. | 0.914 | 0.886 | 0.900 | 0.934 | 0.938 | 0.941 | 0.923 |
| **Use**.  AVE:  CR:  CA: | 0.812  0.928  0.882 | 0.782  0.915  0.860 | 0.786  0.917  0.864 | 0.785  0.916  0.864 | 0.832  0.937  0.899 | 0.792  0.920  0.870 | 0.853  0.946  0.914 |
| I tend to use teleconsulting frequently. | 0.906 | 0.871 | 0.883 | 0.907 | 0.921 | 0.914 | 0.940 |
| I spend a lot of time on teleconsulting. | 0.885 | 0.898 | 0.866 | 0.836 | 0.886 | 0.857 | 0.895 |
| I get involved a lot in teleconsulting. | 0.912 | 0.883 | 0.910 | 0.913 | 0.929 | 0.898 | 0.935 |
| *Note: AVE: Average Variance Extracted ; CR: Composite Reliability ; CA: Cronbach's Alpha* | | | | | | | |

Table 3: Discriminant validity

|  | Behavioural Intention | Effort Expectancy | Facilitating Conditions | Performance Expectancy | Social Influence | Use |
| --- | --- | --- | --- | --- | --- | --- |
| Behavioural Intention | **0.901** | 0.555 | 0.468 | 0.674 | 0.714 | 0.723 |
| Effort Expectancy | 0.623 | **0.865** | 0.739 | 0.707 | 0.500 | 0.412 |
| Facilitating Conditions | 0.528 | 0.847 | **0.840** | 0.566 | 0.436 | 0.333 |
| Performance Expectancy | 0.756 | 0.789 | 0.637 | **0.911** | 0.600 | 0.540 |
| Social Influence | 0.785 | 0.544 | 0.472 | 0.654 | **0.940** | 0.709 |
| Use | 0.812 | 0.455 | 0.365 | 0.600 | 0.777 | **0.901** |
| *Note: Diagonal elements (bold) are the square root of the variance shared between the constructs and their measures (AVE). Under the diagonal elements are the correlations between constructs* (Fornell and Larcker 1981)*. The elements above the diagonal are HTML test values.* | | | | | | |

The objective of analysing the structural model is to determine the paths and R^2^ values. The paths reflect the magnitude of the correlation between the dependent and independent variables, while the R^2^ values demonstrate the proportion of variance explained by each dependent variable in the model. A bootstrapping approach with 10,000 subsamples was employed in this analysis. The results of these measurements are presented in Figure 1. Furthermore, the SRMR was used as a metric of the model's goodness of fit, yielding a value of 0.064, which is lower than the benchmark of 0.08 established by Hair et al. (2019). The combination of the SRMR and R^2^ values suggests that the structural model has a satisfactory explanatory capability.

**Figure 1:** UTAUT global result.

It should be noted that when considering path values, Performance Expectancy and Social Influence are significant predictors of Behavioural Intention. There is a robust correlation between Behavioural Intention and Use. However, Effort Expectancy and Facilitating Conditions do not have a significant impact on either current use or the intention to use non-face-to-face consultations among the full sample of 1412 respondents. These overall results may not accurately depict the complex and diverse behaviours of different segments. Through heterogeneity analysis, we can study these unique behaviours, which may not be revealed in the overall results (Arenas-Gaitán et al. 2019) To uncover these segments, Pathmox analysis will be applied.

**4.1 Pathmox**

Pathmox is an original idea by Gastón Sánchez (Sanchez 2009) to discover heterogeneity in a structural equation model based on decision trees. Decision trees can uncover hidden decision rules and allow high interpretability to explain real applications (Zhao and Nie 2021).

Generally, heterogeneity is associated with a mixture of populations that form differentiated segments (Lamberti, Aluja, and Sanchez 2016). This characteristic between units is a significant problem in data analysis. When a sample is heterogeneous but is treated as homogeneous, the quality of the study can be affected, generating biases in the interpretation (Cataldo, Marino, and Tedesco 2020).

Specifically, Pathmox is oriented to look for differences at the structural level. That is, it searches for segments that differ in the effects between the latent variables of the model. For this purpose, Pathmox uses a recursive algorithm based on binary decision tree learning (Quinlan 1986) to analyse the models associated with different data segments. These data segments are generated by dividing the sample based on additional analysis variables. The analysis variables are external to the structural equation model. Given the objective of Pathmox, the procedure uses the F-test to determine the discrepancy of the true coefficients in two linear regressions associated with different data segments.

The Pathmox algorithm can be summarised as follows. All possible pairs of segments based on the analysis variables are generated, and then the pair of segments with the most significant discrepancy in their effects is selected. Next, a procedure like the previous one is executed for each segment of this pair, and so on, until the differences are not significant, the size of the segments is tiny, or the number of segmentation levels is many. Finally, the algorithm delivers the discovered segments and the variables based on this segmentation. As there are more analysis variables, it is possible to test more potential segment configurations. However, the time complexity of the algorithm increases (Zhao and Nie 2021).

In the present study, seven analysis variables were used to execute the Pathmox: educational level, place of residence, income, age, number of household members under 18 years, and number of elderly household members. The algorithm was run in the R environment (R Development Core Team 2021) using the genpathmox package (Lamberti and Lamberti 2014). The result of the procedure is shown in Figure 2. As can be seen, Pathmox discovered six segments with differences at the structural level, as detailed below.

In general, Pathmox selects the analysis variables age, educational level, and income. Age is the first variable that discriminates. Then, the educational level determines the difference in individuals under 39 years of age. In individuals aged 39 and over, the educational level and income determine this difference. Specifically, the node labelled four comprises 432 individuals between 18 and 38 years old with a K-12 education level. This segment is the largest. Node five has 252 individuals between 18 and 38 years old with a university education level. The node labelled twelve comprises 106 individuals between 39 and 74 years old with a K-12 education level and low income. Node thirteen has 232 individuals between 39 and 74 years old with a K-12 education level and non-low income. The node labelled fourteen is made up of 85 individuals between 39 and 74 years old with a university education level and low income. This segment is the smallest. Finally, node fifteen is formed of 214 individuals between 36 and 74 years old with a university education level and non-low income.

**Figure 2:** Pathmox result.

We calculated the statistic power using G*Power software; the minimum statistic power calculated was 0.764. The 10-times rule (Hair et al., 2011) has been applied, see Table 4.

**Table 4: Estimation of the statistic power.**

|  | **Global** | **Node 4** | **Node 5** | **Node 12** | **Node 13** | **Node 14** | **Node 15** |
| --- | --- | --- | --- | --- | --- | --- | --- |
| Sample size | 1412 | 432 | 252 | 106 | 323 | 85 | 214 |
| 10-times rule | 40 | 40 | 40 | 40 | 40 | 40 | 40 |
| Statistic power | 1.000 | 0.999 | 0.999 | 0.868 | 0.998 | 0.764 | 0.997 |

The main features of the segments are summarised in Table 5, based on the supported relationships in the research model we proposed labels for.

**Table 5: Summary of Pathmox segment characteristics**

| **Node** | **Label** | **Age** | **Educational level** | **Income** | **Differential characteristics compared to the UTAUT global model** |
| --- | --- | --- | --- | --- | --- |
| 4 | Curious Explorers | 18-38 | No University |  | PE->BI significant, but low. |
| 5 | Dynamic Educated | 18-38 | University |  | FC->BI significant. |
| 12 | Experienced Experimenters | 39-74 | No University | Very low | PE->BI not significant. |
| 13 | Resilient Adapters | 39-74 | No University | Low to High | Similar to the global model |
| 14 | Insightful Graduates | 39-74 | University | Low and very low | PE->BI significant, high. |
| 15 | Critical Users | 39-74 | University | Medium to High | FC->USE, significant and negative |

Following the Pathmox results, six segments have been identified (see Table 5). On the one hand, there are two segments made up of young people. The first segment, Curious Explorers (Node 4), comprises young individuals below 39 years of age with no university education and is the largest among the segments. Analysing the teleconsultation adoption process of this segment compared to the rest, we find that it is characterised by performance expectancy (PE) significantly affecting the intention to use this tool (BI) but less so than other segments. This result could be explained by the idea that in a segment made up of young people who are very familiar with new technologies in all aspects of their lives, they may have normalised the advantages of digital media, so their expectations of usefulness are lower when not compared to offline alternatives. These results are also applicable to Node 5. Dynamic Educated (Node 5), the second segment, is made up of young people below 39 years with a university education. This segment, in addition to its significant but low PE to BI ratio (as in Curious Explorers), is particularly characterised by the fact that the facilitating conditions (FC) are significant. In other words, for this segment of university-educated young people, it is important to have suitable devices to carry out medical teleconsultation. This last result may be related to disposable income in this segment. We refer to the fact that these are young people at the beginning of their professional careers and where disposable income is low. The low income in both segments, Curious Explorers and Dynamic Educated (Node 4 and 5), explains why income is not a variable with discriminant power. In both segments, social influence (SI) and perceived usefulness (PE) play a key role in determining the intention and use of teleconsultation.

On the other hand, there are four segments of adults, aged over 39. Experienced Experimenters (Node 12) consists of individuals over 39 years of age with no university education and a perception of lower relative household income compared to their surroundings. In this segment, social influence (SI) is the only variable that shows a strong and significant relationship with the intention to use teleconsultation. Interestingly, this is the only segment where EP does not significantly affect BI. These results can be explained to the extent that for this segment teleconsultation should not be a priority option as a means of contact with the doctor. They would only try to use it if their social environment pushes them to make teleconsultation a regular means of contact with the doctor. Lack of resources, both in terms of devices due to low income levels and skills because of less training, helps to explain this situation. Resilient Adapters (Node 13) is comprised of people over 39 years of age with no university education and a perception of relative household income which is not significantly lower compared to that of their peers. This is one of the largest segments, and both social influence (SI) and perceived usefulness (PE) determine the intention and use of teleconsultation. This is the segment whose technology adoption behaviour is most similar to that of the overall population. Insightful Graduates (Node 14) is the smallest segment, consisting of individuals over 39 years of age with a university education and a perception of lower or much lower relative household income. From the point of view of the process of adopting teleconsultation, this is the segment with the highest explained variances, R2, which suggests a more homogeneous behaviour within the segment. Furthermore, in this segment PE has a significant and very strong relationship with BI. These results indicate that in this segment individuals will be willing to use teleconsultation if they are clearly shown its advantages and benefits. These are university-educated individuals, which implies that they have knowledge and skills but low income levels. Therefore, they will only invest in this type of digital tool if they clearly perceive its usefulness. Finally, Critical Users (Node 15) is formed of individuals over 39 years of age with a university education and a perception of equal or better relative household income. This group is characterised by a significant negative relationship between facilitating conditions and the use of teleconsultation, suggesting that this segment has economic power and access to teleconsultation devices is not an obstacle. These results can be explained by the fact that for this segment, teleconsultation is not their preferred means of interacting with their doctors. These are people with a high purchasing power who are willing to pay the costs for an offline face-to-face consultation.

**4.2. MGA-PLS**

In order to examine the disparities in the uptake of teleconsultation among the six segments determined by the Pathmox analysis, we will carry out a Partial Least Squares-Multi-Group Analysis (PLS-MGA). We will adhere to the guidelines and recommendations outlined by Cheah et al. (2020) and Cheah, Amaro, and Roldán (2023) in conducting this study. The first phase of the procedure involves confirming metric invariance, which can be achieved through conducting a Measurement Invariance Comparison (MICOM) analysis. As our objective is to compare path coefficients between the groups, we will only perform the second step of this test (Latan and Noonan 2017). The results obtained are depicted in Table 6.

**Table 6: MICOM analysis, Step II.**

|  | BI | EE | FC | PE | SI | Use |
| --- | --- | --- | --- | --- | --- | --- |
| N4 vs N5 | 0.954 | 0.321 | 0.848 | 0.108 | 0.284 | 0.075 |
| N12 vs N4 | 0.421 | 0.376 | 0.966 | 0.778 | 0.267 | **0.028** |
| N12 vs N5 | 0.471 | 0.895 | 0.969 | 0.117 | 0.704 | 0.397 |
| N12 vs N13 | 0.132 | 0.593 | 0.61 | 0.082 | 0.677 | 0.612 |
| N12 vs N14 | 0.147 | 0.680 | 0.226 | 0.227 | 0.629 | 0.834 |
| N12 vs N15 | 0.075 | 0.81 | 0.296 | 0.262 | 0.289 | 0.895 |
| N13 vs N4 | 0.546 | 0.078 | 0.431 | **0.009** | **0.008** | **0.005** |
| N13 vs N5 | 0.623 | 0.922 | 0.910 | **0.013** | 0.362 | 0.731 |
| N13 vs N14 | 0.539 | 0.737 | 0.097 | 0.938 | 0.150 | 0.584 |
| N13 vs N15 | 0.109 | 0.45 | 0.161 | 0.895 | 0.577 | 0.575 |
| N14 vs N4 | 0.409 | 0.831 | 0.481 | 0.216 | 0.673 | 0.101 |
| N14 vs N5 | 0.428 | 0.829 | 0.388 | 0.329 | 0.434 | 0.412 |
| N14 vs N15 | 0.070 | 0.627 | 0.915 | 0.814 | 0.067 | 0.892 |
| N15 vs N4 | 0.345 | 0.284 | 0.341 | 0.115 | **0.012** | **0.003** |
| N15 vs N5 | 0.243 | 0.839 | 0.403 | 0.112 | 0.410 | 0.294 |

Appendix 1 presents the outcome of the structural model analysis for each segment individually. The results of the non-parametric PLS-MGA test, which highlights the differences and their significance between segments, are presented in Appendix 2. The data suggests that there are statistically significant disparities in the adoption of telemedicine among the four user groups based on their socio-demographic traits, with significant differences in four out of six cross-segment paths being identified.

In summary, our findings indicate that significant differences exist in 8 out of 15 crossovers between the groups as depicted in Table 6. By integrating the results from both the MICOM Step II analysis (as seen in Table 6) and the PLS-MGA test (in Table 7), we are able to compare the behaviour of the different segments, with the exception of the crossover between N4 and N15. Caution should be exercised when interpreting the significance of the relationship between FC and Use in this particular crossover. Overall, the results highlight disparities in the behaviour of the groups identified by Pathmox.

Table 7: Summary of groups with identified differences.

|  | N4 | N5 | N12 | N13 | N14 | N15 |
| --- | --- | --- | --- | --- | --- | --- |
| N4 |  | No | No | No | No | **Yes** |
| N5 |  |  | **Yes** | No | No | No |
| N12 |  |  |  | **Yes** | **Yes** | **Yes** |
| N13 |  |  |  |  | No | No |
| N14 |  |  |  |  |  | **Yes** |
| N15 |  |  |  |  |  |  |

**5. Discussion of results**

The findings from our study have enabled us to attain our primary research objective of examining the acceptance of teleconsultation technology. This was achieved by identifying and distinguishing between diverse categories of users. In order to delve further into the results obtained, we will use operational objectives as a framework for analysis.

Our first research question was to examine the adoption process of medical teleconsultation using the UTAUT model (Venkatesh et al. 2003). Our findings demonstrate that this was a prudent choice, as the explained variance in our study (0.607 for Behavioural Intention and 0.521 for Use of Teleconsultation) is comparable or greater than in most prior studies on the topic (Harst, Lantzsch, and Scheibe 2019). In other words, our results support the proposed hypothesis H1. Our results contrast with those of other research works on telemedicine (Garavand et al. 2022; Rouidi et al. 2022) and teleconsultation (Alqudah, Al-Emran, and Shaalan 2021) in terms of the impact of facilitating conditions and social influence. In prior works, Facilitating conditions were found to have a significant impact on behavioural intention and usage, whereas in our study, these relationships were not significant. This discrepancy can be attributed to the fact that previous studies (Alqudah et al. 2021; Garavand et al. 2022; Rouidi et al. 2022) focused on health professionals, for whom the work tools serve as facilitating conditions. It is crucial for health systems to make these tools available to professionals for teleconsultation. However, our study took a patient-centric approach and was conducted in a Western country, Spain, where technologies for accessing teleconsultation (e.g., computers, tablets, or smartphones) are widely available. This difference in perspective may explain the divergence in our results. Alternatively, in prior research collected from various literature reviews (Alqudah et al. 2021; Garavand et al. 2022; Rouidi et al. 2022), the impact of social influence on behavioural intention has not been found to be significant. However, in our findings, social influence emerged as the variable with the greatest effect on intention to use teleconsultation. This discrepancy could be attributed to several factors, including the differences between healthcare professionals and patients as well as the timing of previous research. Most of the literature was conducted prior to or during the COVID-19 confinement period, when teleconsultation was not a widespread practice in many healthcare systems. As such, patients' social environments were not well-versed in the use of telemedicine. The COVID-19 pandemic served as a catalyst for the widespread adoption of teleconsultation globally, particularly in Western economies. Our research was conducted in Spain between May and November 2022, two years after the end of lockdown and with the majority of the population vaccinated. In this new post-pandemic era, most Western citizens have some degree of familiarity with teleconsultation, which explains the heightened influence of social factors on individuals' teleconsultation behaviours.

The second research question focused on distinguishing segments of medical teleconsultation users with varying acceptance behaviours, determined by socio-demographic characteristics including age, educational background, and income level. Our results demonstrate the importance of socio-demographic characteristics in explaining differences in the teleconsultation acceptance process. In this sense, we see that age is the variable that discriminates first. The cut-off point that makes the difference is 39 years of age. We can interpret these results, taking into account the family life cycle (Shannon et al. 2020), where we move from a phase characterised by more individualistic behaviour in the case of individuals or young families with few members, to a new situation where there is more collective behaviour in the case of older people included in families with a larger number of members, whether ascendants or descendants. The second most important variable is education, specifically having a university education. We understand that these studies improve perceptions such as the usefulness or ease of using medical teleconsultation. Finally, the third variable that helps us to distinguish groups by their behaviour is the relative perception of family income. This variable does not affect younger groups (nodes 4 and 5) and only affects older groups (nodes 12, 13, 14 and 15). Moreover, it is very interesting how this variable offers different cut-offs depending on education. For those with a university education, the cut-off is at the perception of equal or higher (three out of five). This results in a small group of older, university-educated respondents who perceive their income as lower than their peers. This is consistent with the data we have on the Spanish society. Having a university education is a good predictor of individuals' incomes (Sempere and Calatayud 2022). For this reason, in the case of non-university respondents, the groups are divided between those who have a perception that their household income is much worse than that of their peers (one out of five), and the rest. In summary, the results lend support to the proposed hypothesis H2, which posits that the combination of socio-demographic characteristics, such as age, educational level or income level, of users of medical teleconsultation services gives rise to statistically differentiated segments in terms of their technology acceptance processes. In particular, the Pathmox analysis based on socio-demographic variables has revealed six patient segments with distinct behaviours in telemedicine acceptance, emphasising that socio-demographic characteristics play a crucial role in explaining individual behaviour in teleconsultation. Our results show how socio-demographic characteristics have an impact on the behaviour of individuals and families towards telemedicine. However, according to our results, the importance of socio-demographic characteristics may vary, as with the perception of relative income, from one circumstance to another.

Finally, we analyse the similarities and differences between the different segments obtained above with respect to their process of acceptance of teleconsultation. Although significant differences have been identified among certain segments, the majority of these differences are observed between segments Experienced Experimenters and Critical Users (N12 and N15), i.e., those over 39 years old without a university education and with a perception of a lower relative household income compared to their peers and those over 39 years old with a university education and a perception of a relative household income equal to or better than their peers. These two segments have a more distinct behaviour towards teleconsultation adoption compared to the others. For the Experienced Experimenters, most of the differences are seen in the relationship between perceived usefulness and intention to use teleconsultation, with this group having little regard for the usefulness of teleconsultation. In the case of Critical Users, the differences are associated with facilitating conditions, as this group is less inclined to use teleconsultation despite having the necessary technology available. On the other hand, no differences were found between young individuals regarding teleconsultation adoption. In conclusion, when designing strategies to integrate teleconsultation into healthcare systems, it is essential to consider the distinct adoption processes among the four detected groups, necessitating the development of specific strategies for each group that take into account their unique characteristics.

Our findings align with those of other researchers who have employed user segmentation in the health sector. For example, Scheufele et al. (2022) employ a generic segmentation strategy comprising 68 US consumer groups, delineated by a consultancy firm for a diverse array of products. These groups are characterised by a combination of socio-demographic, lifestyle, behavioural, attitudinal, and habit information. The authors employ this approach to identify segments with an elevated risk of depression. In the context of mental health, Bloem et al. (2020) report an a priori segmentation of four segments based on two variables: acceptance and perceived control of their health. Furthermore, the authors utilise socio-demographic variables to characterise the identified segments. A comparison of the results reveals similarities between the S1 segment (high acceptance of health and high perceived control) and the Critical Users segment, which is characterised by a university education and high income levels. Additionally, their S4 segment (characterised by low acceptance and low control) is analogous to our Resilient Adapters segment, comprising older individuals without university education. In the domain of health systems management, the research conducted by Vuik, Mayer, and Darzi (2016) and Brommels (2020) on the UK health system is particularly noteworthy. In both cases, the segmentation is developed on the basis of population health criteria. In the segmentation proposed by Vuik et al. (2016), the age criterion is also a significant factor, with the resulting classification aligning with our findings. The authors identify three segments of patients with a mean age below 38 years, and five other groups with mean ages above the specified cut-off point. Brommels (2020) identifies seven patient segments based on their health status, four of which can be primarily served by digital health systems, such as teleconsultation. In a study conducted in South Korea, Shin and Yun (2011) identified four distinct groups of individuals who seek medical information through digital means. In accordance with our findings, the researchers conclude that educational attainment and income level are useful criteria for segmenting the population in order to target these types of services. In a recent study, Lakoma et al. (2024) conducted a segmentation of patients of digital healthcare services in Finland. A distinction is drawn between patients who attend face-to-face medical visits and those who utilise digital means, such as teleconsultation. The socio-demographic profile of their segment of occasional digital clinic patients is comparable to that of our Curious Explorers cohort, comprising young individuals without a university education.

**6. Theoretical, Management and Social Contributions**.

From a scientific and theoretical perspective, our study adds to the works of Rouidi et al. (2022) and Garavand et al. (2022) by taking a patient-centred approach to examining the medical technology acceptance process. While these previous studies focus on the views of practitioners, our research highlights the differences that exist between the perspectives of patients and professionals. For instance, while facilitating conditions are deemed crucial from a professional viewpoint (Garavand et al. 2022; Rouidi et al. 2022), our findings do not support this notion from the patient's perspective. Conversely, our results demonstrate that social influence is a crucial factor in determining the adoption of medical teleconsultation technology from the patient's point of view, which is in stark contrast to the limited importance placed on it by professionals. Our research is significant as it is one of the few studies to adopt a patient-centric approach, which is particularly important given that most of the existing literature on medical teleconsultation has been analysed from the professional's perspective (Alqudah et al. 2021). Additionally, our study is also noteworthy as it is the first research found in the literature that segments patients in relation to the adoption process of medical teleconsultation technology, revealing important differences in behaviour among different patient segments.

Our research provides a comprehensive overview of the various segments involved in the process of adopting teleconsultation from the perspective of managers. By examining these segments and their unique characteristics, we aim to help managers of healthcare-related companies, such as health insurance providers and hospitals, effectively integrate teleconsultation tools into their service offerings. For instance, for Curious Explorers and Dynamic Educated, it would be appropriate for campaigns to emphasise how teleconsultation is integrated into young people's lifestyles, coexisting with other digital tools with which they may be more familiar. For Experienced Experimenters, campaigns are proposed pointing out that teleconsultation is a widespread tool, available to everyone. To address Insightful Graduates, we propose campaigns pointing out especially the advantages and benefits of teleconsultation over traditional medical consultation. To do so, some arguments could be the time savings and the prevention of contagions in the waiting rooms of medical centres. Finally, for Critical Users, our findings reveal a negative relationship between facilitating conditions and teleconsultation usage among individuals aged 39 and above, with a university education and higher perceived relative household income. This result is due to these individuals' preference for face-to-face consultations, despite their ability to use technology as a result of their good economic status. When devising a strategy for this segment, it would be wise to highlight the complementary nature of teleconsultation with traditional face-to-face consultations, stressing its benefits in contexts that do not require an in-person visit to a medical centre, such as clinical analysis explanations.

Finally, our research has important social implications. Medical teleconsultation can play a crucial role in maintaining the viability of healthcare systems. Our findings indicate that the social context of individuals is a key factor in their adoption of this technology. Given that we now inhabit a post-COVID world where many individuals have had experience with teleconsultation during periods of quarantine and social distancing, it may be beneficial to highlight the successes of this tool in specific medical services or for certain patient groups. This could help to enhance the image of teleconsultation and promote its integration into healthcare services, particularly for consultations that do not require an in-person visit to a medical centre.

**7. Conclusions, limitations and future research.**

The permanent integration of digital technologies into healthcare systems is a reality. In this scenario, medical teleconsultation plays a crucial role in ensuring the sustainability of the Health System. However, for this digital tool to be effectively integrated into the Health System, it is imperative to consider not only the perspective of healthcare professionals, but also that of the patients. Our research has identified six distinct segments of medical teleconsultation patients, each with unique socio-demographic characteristics that impact their adoption of this tool.

Our study has certain limitations that can provide the basis for future research efforts. Firstly, our research is carried out in Spain, where the public health system has significant influence compared to the private system. It would be of interest to conduct a similar study in countries where the private health system has a larger presence. Secondly, we employed PLS-SEM as a statistical method, which focuses on linear cause-and-effect relationships. Further research utilising non-linear methods could prove valuable. Thirdly, one limitation of this study relates to the use of convenience sampling, and though our post-hoc analysis indicates a slight underestimation of proportions in our survey compared to the true population values, suggesting that while it should be considered in sensitive analyses or policy decisions, this may not significantly alter the overall interpretation of the study findings. Finally, our data have a distinct cross-sectional nature, only depicting the current reality at a given moment in time. It would be beneficial to conduct longitudinal studies in the future to track the development of the adoption process over time.

**8. References**

Adeghe, Ehizogie Paul, Chioma Anthonia Okolo, and Olumuyiwa Tolulope Ojeyinka. 2024. “A Review of Emerging Trends in Telemedicine: Healthcare Delivery Transformations.” *International Journal of Life Science Research Archive* 6(1):137–47. doi: 10.53771/ijlsra.2024.6.1.0040.

Albahri, Ahmed Shihab, Jwan K. Alwan, Zahraa K. Taha, Sura F. Ismail, Rula A. Hamid, A. A. Zaidan, Osamah Shihab Albahri, B. B. Zaidan, Abdullah Hussein Alamoodi, and M. A. Alsalem. 2021. “IoT-Based Telemedicine for Disease Prevention and Health Promotion: State-of-the-Art.” *Journal of Network and Computer Applications* 173:102873.

Alqudah, Adi A., Mostafa Al-Emran, and Khaled Shaalan. 2021. “Technology Acceptance in Healthcare: A Systematic Review.” *Applied Sciences (Switzerland)* 11(22):11. doi: 10.3390/app112210537.

Arenas-Gaitán, Jorge, A. Francisco Villarejo Ramos, and Begoña Peral-Peral. 2019. “A Posteriori Segmentation of Elderly Internet Users: Applying PLS-POS.” *Marketing Intelligence and Planning* 38(3):340–53. doi: 10.1108/MIP-01-2019-0057.

Augusterfer, Eugene F., Colleen R. O’Neal, Shannon W. Martin, Taiwo Lateef Sheikh, and Richard F. Mollica. 2020. “The Role of Telemental Health, Tele-Consultation, and Tele-Supervision in Post-Disaster and Low-Resource Settings.” *Current Psychiatry Reports* 22(12):1–10.

Banning, Stefan, Marc Höglinger, Delia Meyer, and Oliver Reich. 2022. “Evaluation of the Effect of a Multifunctional Telemedicine Device on Health Care Use and Costs: A Nonrandomized Pragmatic Trial.” *Telemedicine and E-Health*.

Baudier, P., G. Kondrateva, C. Ammi, V. Chang, and F. Schiavone. 2022. “Digital Transformation of Healthcare during the COVID-19 Pandemic: Patients’ Teleconsultation Acceptance and Trusting Beliefs.” *Technovation*. doi: https://doi.org/10.1016/j.technovation.2022.102547.

Baudier, Patricia, Galina Kondrateva, Chantal Ammi, Victor Chang, and Francesco Schiavone. 2021. “Patients’ Perceptions of Teleconsultation during COVID-19: A Cross-National Study.” *Technological Forecasting and Social Change* 163:120510. doi: 10.1016/J.TECHFORE.2020.120510.

Bhatnagar, Amit, and Sanjoy Ghose. 2004. “A Latent Class Segmentation Analysis of E-Shoppers.” *Journal of Business Research* 57(7):758–67. doi: 10.1016/S0148-2963(02)00357-0.

Binesh, Fatemeh, and Seyhmus Baloglu. 2023. “Are We Ready for Hotel Robots after the Pandemic? A Profile Analysis.” *Computers in Human Behavior* 147:107854. doi: 10.1016/J.CHB.2023.107854.

Blanco Terés, Lara, Carlos Cerdán Santacruz, Javier García Septiem, Rocío Maqueda González, José María Lopesino González, Alba Correa Bonito, and Elena Martín-Pérez. 2022. “Patients’ Perceived Satisfaction through Telephone-Assisted Tele-Consultation during the SARS-CoV-2 Pandemic Period: Observational Single-Centre Study at a Tertiary-Referral Colorectal Surgery Department.” *Surgical Innovation* 29(1):35–43.

Bloem, Sjaak, Joost Stalpers, Edward A. G. Groenland, Kees van Montfort, W. Fred van Raaij, and Karla de Rooij. 2020. “Segmentation of Health-Care Consumers: Psychological Determinants of Subjective Health and Other Person-Related Variables.” *BMC Health Services Research* 20(1):726. doi: 10.1186/s12913-020-05560-4.

Bockshecker, Alina, Sarah Hackstein, and Ulrike Baumöl. 2018. “Systematization of the Term Digital Transformation and Its Phenomena from a Socio-Technical Perspective – A Literature Review.” in *Research Papers ECIS 2018 Proceedings*. AIS Electronic Library (AISeL).

Brommels, Mats. 2020. “Patient Segmentation: Adjust the Production Logic to the Medical Knowledge Applied and the Patient’s Ability to Self-Manage—A Discussion Paper.” *Frontiers in Public Health* 8. doi: 10.3389/fpubh.2020.00195.

Cataldo, Rosanna, Marina Marino, and N. Tedesco. 2020. “PLS-PM: New Methodological Developments for Constructing Model Based Social Indicators.” Pp. 39–60 in *Partial Least Squares. Path Modeling for studying Social Issue*, edited by E. Amaturo, M. Grassia, and C. Carlo Lauro. Milan: Mc Graw Hill.

Cheah, Jun Hwa, Suzanne Amaro, and José L. Roldán. 2023. “Multigroup Analysis of More than Two Groups in PLS-SEM: A Review, Illustration, and Recommendations.” *Journal of Business Research* 156:113539. doi: 10.1016/j.jbusres.2022.113539.

Cheah, Jun Hwa, Ramayah Thurasamy, Mumtaz Ali Memon, Francis Chuah, and Hiram Ting. 2020. “Multigroup Analysis Using Smartpls: Step-by-Step Guidelines for Business Research.” *Asian Journal of Business Research* 10(3):I–XIX.

Chellaiyan, Vinoth G., A. Y. Nirupama, and Neha Taneja. 2019. “Telemedicine in India: Where Do We Stand?” *Journal of Family Medicine and Primary Care* 8(6):1872.

Chimento-Díaz, Sara, Pablo Sánchez-García, Cristina Franco-Antonio, Esperanza Santano-Mogena, Isabel Espino-Tato, and Sergio Cordovilla-Guardia. 2022. “Factors Associated with the Acceptance of New Technologies for Ageing in Place by People over 64 Years of Age.” *International Journal of Environmental Research and Public Health* 19(5):2947. doi: 10.3390/ijerph19052947.

Chua, SL, D. Chem, and P. Wong. 1999. “Computer Anxiety and Its Correlates: A Meta Analysis.” *Computers in Human Behavior* 14:559–77. doi: doi.org/10.1016/S0747-5632(99)00039-4.

Creaser, Amy V., Jennifer Hall, Silvia Costa, Daniel D. Bingham, and Stacy A. Clemes. 2022. “Exploring Families’ Acceptance of Wearable Activity Trackers: A Mixed-Methods Study.” *International Journal of Environmental Research and Public Health* 19(6). doi: 10.3390/ijerph19063472.

Dahlgren, C., E. Spånberg, S. Sveréus, M. Dackehag, P. Wändell, and C. Rehnberg. 2024. “Short-and Intermediate-Term Impact of DTC Telemedicine Consultations on Subsequent Healthcare Consumption.” *The European Journal of Health Economics* 25(1):157–76. doi: https://doi.org/10.1007/s10198-023-01572-z.

Davis, F. D. 1985. “A Technology Acceptance Model for Empirically Testing New End-User Information Systems: Theory and Results.”

Davis, Fred D. 1989. “Perceived Usefulness, Perceived Ease of Use, and User Acceptance of Information Technology.” *MIS Quarterly* 13(3):319–40. doi: 10.2307/249008.

Davis, Fred D., Richard P. Bagozzi, and Paul R. Warshaw. 1989. “User Acceptance of Computer Technology: A Comparison of Two Theoretical Models.” *Management Science* 35(8):982–1003. doi: 10.1287/mnsc.35.8.982.

European Commision. 2024a. “ EU Health Policy. Overview.” *Https://Health.Ec.Europa.Eu/Eu-Health-Policy/Overview_en#priorities-for-2021-2027*. Retrieved September 20, 2024 (https://health.ec.europa.eu/eu-health-policy/overview_en#priorities-for-2021-2027).

European Commision. 2024b. *Factsheet on European Health Data Space*. Brussels.

Farivar, S., M. Abouzahra, and M. Ghasemaghaei. 2020. “Wearable Device Adoption among Older Adults: A Mixed-Methods Study.” *International Journal of Information Management* 55. doi: 10.1016/j.ijinfomgt.2020.102209.

Fornell, Claes, and David F. Larcker. 1981. “Evaluating Structural Equation Models with Unobservable Variables and Measurement Error.” *Journal of Marketing Research* 18(1):39–50.

Garattini, Livio, Marco Badinella Martini, and Michele Zanetti. 2021. “More Room for Telemedicine after COVID-19: Lessons for Primary Care?” *European Journal of Health Economics* 22(2):183–86. doi: 10.1007/S10198-020-01248-Y.

Garavand, Ali, Nasim Aslani, Hamed Nadri, Saeideh Abedini, and Shirin Dehghan. 2022. “Acceptance of Telemedicine Technology among Physicians: A Systematic Review.” *Informatics in Medicine Unlocked* 30. doi: 10.1016/j.imu.2022.100943.

Gradillas, Maria, and Llewellyn D. W. Thomas. 2023. “Distinguishing Digitization and Digitalization: A Systematic Review and Conceptual Framework.” *Journal of Product Innovation Management*. doi: 10.1111/jpim.12690.

Hair, Joseph F., Jeffrey J. Risher, Marko Sarstedt, and Christian M. Ringle. 2019. “When to Use and How to Report the Results of PLS-SEM.” *European Business Review* 31(1):2–24.

Harst, Lorenz, Hendrikje Lantzsch, and Madlen Scheibe. 2019. “Theories Predicting End-User Acceptance of Telemedicine Use: Systematic Review.” *Journal of Medical Internet Research* 21(5). doi: 10.2196/13117.

Henseler, Jörg, Christian M. Ringle, and Marko Sarstedt. 2015. “A New Criterion for Assessing Discriminant Validity in Variance-Based Structural Equation Modeling.” *Journal of the Academy of Marketing Science* 43(1):115–35.

Hernández-Zambrano, S. M., R. A. Castiblanco-Montañez, P. M. Á, Guerrero, M. A. G. Moreno, L. G. M. ;. Sacristán, G. S. Rodríguez-Vargas, L. Villareal, J. A. ;. Rubio, E. Quintero, M. A. Gómez, J. Chávez-Chávez, D. P. R. Triana, R. Casanova, A. Rojas-Villarraga, and P. Santos-Moreno. 2021. “Experience of Tele Consulting Care for Health Care Professionals and Patients with Rheumatoid Arthritis, in the Health Emergency Due to Covid-19.” *Journal of Clinical Rheumatology*.

Kavandi, Hamidreza, and Mirou Jaana. 2020. “Factors That Affect Health Information Technology Adoption by Seniors: A Systematic Review.” *Health and Social Care in the Community* 28(6):1827–42. doi: 10.1111/hsc.13011.

Kock, Ned. 2015. “Common Method Bias in PLS-SEM: A Full Collinearity Assessment Approach.” *International Journal of E-Collaboration* 11(4):1–10. doi: 10.4018/ijec.2015100101.

Kraus, Sascha, Francesco Schiavone, Anna Pluzhnikova, and Anna Chiara Invernizzi. 2021. “Digital Transformation in Healthcare: Analyzing the Current State-of-Research.” *Journal of Business Research* 123:557–67. doi: 10.1016/j.jbusres.2020.10.030.

Kwon, Ohbyung, and Yixing Wen. 2010. “An Empirical Study of the Factors Affecting Social Network Service Use.” *Computers in Human Behavior* 26(2):254–63.

Lakoma, Sanna, Henna Pasanen, Kaisla Lahdensuo, Jaakko Pehkonen, Jutta Viinikainen, and Paulus Torkki. 2024. “Quality of the Digital Gp Visits and Characteristics of the Users: Retrospective Observational Study.” *Scandinavian Journal of Primary Health Care* 1–9. doi: 10.1080/02813432.2024.2380921.

Lamberti, G., and M. G. Lamberti. 2014. “Genpathmox.”

Lamberti, Giuseppe, Tomas Banet Aluja, and Gaston Sanchez. 2016. “The Pathmox Approach for PLS Path Modeling Segmentation.” *Applied Stochastic Models in Business and Industry* 32(4). doi: 10.1002/asmb.2168.

Lamberti, Giuseppe, Tomas Banet-Aluja, and Gaston Sanchez. 2017. “The Pathmox Approach for PLS Path Modeling Segmentation.” *Applied Stochastic Models in Business and Industry* 32(6):453–68. doi: 10.1002/asmb.2168.

Latan, H., and R. Noonan. 2017. *Partial Least Squares Path Modeling. Basic Concepts, Methodological Issues and Applications*. Springer International Publishing.

Lindsay, J. S. B. 1968. “Types of Family and Family Types.” *Family Process* 7(1):51–66. doi: 10.1111/j.1545-5300.1968.00051.x.

Liu, C., K. Hung, D. Wang, and S. Wang. 2020. “Determinants of Self-Service Technology Adoption and Implementation in Hotels: The Case of China.” *Journal of Hospitality Marketing and Management* 29(6):636–61. doi: 10.1080/19368623.2020.1689216.

Liu, Chunfeng, Renee Lim, Silas Taylor, and Rafael A. Calvo. 2019. “Students’ Behavioural Engagement in Reviewing Their Tele-Consultation Feedback within an Online Clinical Communication Skills Platform.” *Computers in Human Behavior* 94:35–44.

Lopez, Ana Maria, Kenneth Lam, and Ramya Thota. 2021. “Barriers and Facilitators to Telemedicine: Can You Hear Me Now?” *American Society of Clinical Oncology Educational Book* 41:25–36.

Lu, Wei, Dongle Wei, Changze Li, Pan Gao, Rui Ma, Yunkai Zhai, and Chengzeng Wang. 2024. “How to Promote Telemedicine Patient Adoption Behavior for Greener Healthcare?” *Journal of Cleaner Production* 434:139884. doi: 10.1016/J.JCLEPRO.2023.139884.

Majcherek, Dawid, Scott William Hegerty, Arkadiusz Michał Kowalski, Małgorzata Stefania Lewandowska, and Desislava Dikova. 2024. “Opportunities for Healthcare Digitalization in Europe: Comparative Analysis of Inequalities in Access to Medical Services.” *Health Policy* 139:104950. doi: 10.1016/j.healthpol.2023.104950.

Makhni, Melvin C., Grant J. Riew, and Marissa G. Sumathipala. 2020. “Telemedicine in Orthopaedic Surgery: Challenges and Opportunities.” *JBJS* 102(13):1109–15.

Matthews, Lucy M., Marko Sarstedt, Joseph F. Hair, and Christian M. Ringle. 2016. “Identifying and Treating Unobserved Heterogeneity with FIMIX-PLS.” *European Business Review* 28(2):208–24. doi: 10.1108/EBR-09-2015-0095.

Mazzuca, Daniela, Massimiliano Borselli, Santo Gratteri, Giovanna Zampogna, Alessandro Feola, Marcello Della Corte, Francesca Guarna, Vincenzo Scorcia, and Giuseppe Giannaccare. 2022. “Applications and Current Medico-Legal Challenges of Telemedicine in Ophthalmology.” *International Journal of Environmental Research and Public Health* 19(9):5614.

Mishkin, Adrienne D., Stephanie Cheung, Justin Capote, Weijia Fan, and Philip R. Muskin. 2022. “Survey of Clinician Experiences of Telepsychiatry and Tele-Consultation-Liaison Psychiatry.” *Journal of the Academy of Consultation-Liaison Psychiatry* 63(4):334–44.

Nittari, Giulio, Ravjyot Khuman, Simone Baldoni, Graziano Pallotta, Gopi Battineni, Ascanio Sirignano, Francesco Amenta, and Giovanna Ricci. 2020a. “Telemedicine Practice: Review of the Current Ethical and Legal Challenges.” *Telemedicine and E-Health* 26(12):1427–37.

Nittari, Giulio, Ravjyot Khuman, Simone Baldoni, Graziano Pallotta, Gopi Battineni, Ascanio Sirignano, Francesco Amenta, and Giovanna Ricci. 2020b. “Telemedicine Practice: Review of the Current Ethical and Legal Challenges.” *Telemedicine and E-Health* 26(12):1427–37.

Paas, F., and J. J. G. van Merriënboer. 2020. “Cognitive-Load Theory: Methods to Manage Working Memory Load in the Learning of Complex Tasks.” *Current Directions in Psychological Science* 29(4):394–98. doi: 10.1177/0963721420922183.

Porter, Constance Elise, and Naveen Donthu. 2006. “Using the Technology Acceptance Model to Explain How Attitudes Determine Internet Usage: The Role of Perceived Access Barriers and Demographics.” *Journal of Business Research* 59(9):999–1007. doi: 10.1016/j.jbusres.2006.06.003.

Quinlan, J. R. 1986. “Induction of Decision Trees.” *Machine Learning* 1(1):81–106. doi: 10.1007/bf00116251.

R Development Core Team. 2021. “R: A Language and Environment for Statistical Computing.” *R Foundation for Statistical Computing*.

Rouidi, Mohammed, Abd Elmajid Elouadi, Amine Hamdoune, Khadija Choujtani, and Adam Chati. 2022. “TAM-UTAUT and the Acceptance of Remote Healthcare Technologies by Healthcare Professionals: A Systematic Review.” *Informatics in Medicine Unlocked* 32. doi: 10.1016/j.imu.2022.101008.

Sageena, Geetanjali, Munish Sharma, and Ajita Kapur. 2021. “Evolution of Smart Healthcare: Telemedicine During COVID-19 Pandemic.” *Journal of The Institution of Engineers (India): Series B* 102(6):1319–24. doi: 10.1007/s40031-021-00568-8.

Sanchez, G. 2009. “Pathmox Approach: Segmentation Trees in Partial Least Squares Path Modeling .” Universitat Politècnica de Catalunya, Barcelona.

Sarstedt, Marko. 2008. “A Review of Recent Approaches for Capturing Heterogeneity in Partial Least Squares Path Modelling.” *Journal of Modelling in Management* 3(2):140–61. doi: 10.1108/17465660810890126.

Sarstedt, Marko, Christian M. Ringle, and Joseph F. Hair. 2017a. “Partial Least Squares Structural Equation Modeling.” Pp. 1–40 in *Handbook of Market Research*.

Sarstedt, Marko, Christian M. Ringle, and Joseph F. Hair. 2017b. “Treating Unobserved Heterogeneity in PLS-SEM.” Pp. 361–81 in *Partial Least Squares Path Modeling*.

Scheufele, Elisabeth, Brandi Hodor, George Popa, Suwei Wang, and William Kassler. 2022. “Population Segmentation Using a Novel Socio-Demographic Dataset.” *Online Journal of Public Health Informatics* 14(1). doi: 10.5210/ojphi.v14i1.11651.

Schönauer, A. L., and S. Glanz. 2022. “Hydrogen in Future Energy Systems: Social Acceptance of the Technology and Its Large-Scale Infrastructure.” *International Journal of Hydrogen Energy* 47(24):12251–63. doi: 10.1016/j.ijhydene.2021.05.160.

Schuetz, S., and V. Venkatesh. 2020. “Blockchain, Adoption, and Financial Inclusion in India: Research Opportunities.” *International Journal of Information Management* 52. doi: 10.1016/j.ijinfomgt.2019.04.009.

Sempere, Mónica Martí, and Carmen Ródenas Calatayud. 2022. “Grants Programme and Tuition Fees Policy in the Spanish Public University System: Is It Really Effective?” *Revista de Educacion* 2022(398):135–60. doi: 10.4438/1988-592X-RE-2022-398-555.

Shannon, Randall, Thuckavadee Sthienrapapayut, George P. Moschis, Thorsten Teichert, and Betul Balikcioglu. 2020. “Family Life Cycle and the Life Course Paradigm: A Four-Country Comparative Study of Consumer Expenditures.” *Journal of Global Scholars of Marketing Science* 30(1):34–44. doi: 10.1080/21639159.2019.1613913.

Sharma, Rahul. 2013. “The Family and Family Structure Classification Redefined for the Current Times.” *Journal of Family Medicine and Primary Care* 2(4):306. doi: 10.4103/2249-4863.123774.

Shin, Sung Hee, and Eun Kyoung Yun. 2011. “Understanding the Online Health Information User Profiles in Korea: From a Psychological Perspective.” *Telemedicine and E-Health* 17(5):341–47. doi: 10.1089/tmj.2010.0170.

Soper, D. S. 2023. “A-Priori Sample Size Calculator for Structural Equation Models.” *Https://Www.Danielsoper.Com/Statcalc*.

Tsertsidis, Antonios, Ella Kolkowska, and Karin Hedström. 2019. “Factors Influencing Seniors’ Acceptance of Technology for Ageing in Place in the Post-Implementation Stage: A Literature Review.” *International Journal of Medical Informatics* 129:324–33. doi: 10.1016/j.ijmedinf.2019.06.027.

Venkatesh, Morris, Davis, and Davis. 2003. “User Acceptance of Information Technology: Toward a Unified View.” *MIS Quarterly* 27(3):425. doi: 10.2307/30036540.

Venkatesh, V., M. G. Morris, G. B. Davis, and F. D. Davis. 2003. “User Acceptance of Information Technology: Toward a Unified View.” *Mis Quarterly* 27(3):425–78.

Venkatesh, V., JYL. Thong, and X. Xu. 2012. “Consumer Acceptance and Use of Information Technology: Extending the Unified Theory of Acceptance and Use of Technology.” *MIS Quarterly* 36(1):157–78.

Villarejo-Ramos, Ángel F., Begoña Peral-Peral, and Jorge Arenas-Gaitán. 2019. “Latent Segmentation of Older Adults in the Use of Social Networks and E-Banking Services.” *Information Research* 24(4).

Vranda, Mysore Narasimha, and Vasanthra Radhakrishnan Cicil. 2022. “Tele-Consultation for the Survivors of Intimate Partner Violence: Guidelines for Mental Health Professionals.” *Indian Journal of Psychological Medicine* 44(4):349–53.

Vuik, Sabine I., Erik Mayer, and Ara Darzi. 2016. “A Quantitative Evidence Base for Population Health: Applying Utilization-Based Cluster Analysis to Segment a Patient Population.” *Population Health Metrics* 14(1):44. doi: 10.1186/s12963-016-0115-z.

Vural, Sevilay, and Hayri Ramadan. 2019a. “A Short Review on the Comparison of Consultation Systems and Tools in the Emergency Department Practice: Tele-Consultation.” *Cumhuriyet Medical Journal* 41(2):239–43.

Vural, Sevilay, and Hayri Ramadan. 2019b. “A Short Review on the Comparison of Consultation Systems and Tools in the Emergency Department Practice: Tele-Consultation.” *Cumhuriyet Medical Journal* 41(2):239–43.

Westland, Ch. J. 2010. “Lower Bounds on Sample Size in Structural Equation Modeling.” *Electronic Commerce Research and Applications* 9(6):476–87. doi: 10.1016/J.ELERAP.2010.07.003.

Wright, Jane, and Michelle Honey. 2016. “NEW ZEALAND NURSES’EXPERIENCE OF TELE-CONSULTATION WITHIN SECONDARY AND TERTIARY SERVICES TO PROVIDE CARE AT A DISTANCE.” *Nursing Praxis in New Zealand* 32(2).

Yang, Hongze, Xitong Guo, Zeyu Peng, and Kee-Hung Lai. 2022. “Patient Empowerment in an Online Health Platform: Exploring the Quadratic Effects of Patients’ Conscious-Competence on Perceived Health Status.” *Computers in Human Behavior* 107346. doi: 10.1016/j.chb.2022.107346.

Zhao, Xin, and Xiaokai Nie. 2021. “Splitting Choice and Computational Complexity Analysis of Decision Trees.” *Entropy* 23(10). doi: 10.3390/e23101241.

**9. Appendix (continued) en vez de (continue) :-**

Appendix 1: Paths by Segments

|  | Global | | N12 | | N13 | | N14 | | N15 | | N4 | | N5 | |
| --- | --- | --- | --- | --- | --- | --- | --- | --- | --- | --- | --- | --- | --- | --- |
|  | Path | P-value | Path | P-value | Path | P-value | Path | P-value | Path | P-value | Path | P-value | Path | P-value |
| BI -> Use | **0.726** | **0.000** | **0.667** | **0.000** | **0.753** | **0.000** | **0.708** | **0.000** | **0.766** | **0.000** | **0.713** | **0.000** | **0.743** | **0.000** |
| EE -> BI | 0.068 | 0.060 | 0.240 | 0.348 | 0.099 | 0.211 | 0.077 | 0.445 | -0.015 | 0.854 | 0.089 | 0.117 | 0.074 | 0.390 |
| FC -> BI | 0.026 | 0.366 | -0.172 | 0.386 | -0.006 | 0.929 | -0.050 | 0.504 | 0.120 | 0.057 | -0.031 | 0.586 | **0.103** | **0.050** |
| FC -> Use | -0.007 | 0.742 | 0.105 | 0.158 | -0.022 | 0.594 | 0.136 | 0.115 | **-0.113** | **0.022** | 0.008 | 0.819 | -0.059 | 0.303 |
| PE -> BI | **0.328** | **0.000** | 0.061 | 0.615 | **0.392** | **0.000** | **0.598** | **0.000** | **0.390** | **0.000** | **0.278** | **0.000** | **0.289** | **0.001** |
| SI -> BI | **0.472** | **0.000** | **0.652** | **0.000** | **0.430** | **0.000** | **0.334** | **0.000** | **0.475** | **0.000** | **0.514** | **0.000** | **0.439** | **0.000** |
| R2 |  |  |  |  |  |  |  |  |  |  |  |  |  |  |
| BI | 0.607 | | 0.529 | | 0.652 | | 0.725 | | 0.715 | | 0.553 | | 0.587 | |
| Use | 0.521 | | 0.519 | | 0.549 | | 0.618 | | 0.513 | | 0.512 | | 0.516 | |

Appendix 2: Test PLS-MGA

|  | N12 - N13 | | N12 - N14 | | N12 - N15 | | N12 - N4 | | N12 - N5 | | N13 - N14 | | N13 - N15 | | N13 - N4 | | N13 - N5 | | N14 - N15 | |
| --- | --- | --- | --- | --- | --- | --- | --- | --- | --- | --- | --- | --- | --- | --- | --- | --- | --- | --- | --- | --- |
|  | Diff | P-val. | Diff | P-val. | Diff | P-val. | Diff | P-val. | Diff | P-val. | Diff | P-val. | Diff | P-val. | Diff | P-val. | Diff | P-val. | Diff | P-val. |
| BI -> Use | -0.086 | 0.292 | -0.041 | 0.675 | -0.099 | 0.206 | -0.046 | 0.551 | -0.076 | 0.365 | 0.045 | 0.598 | -0.013 | 0.828 | 0.040 | 0.441 | 0.010 | 0.892 | -0.058 | 0.478 |
| EE -> BI | 0.141 | 0.601 | 0.163 | 0.562 | 0.255 | 0.382 | 0.152 | 0.574 | 0.166 | 0.551 | 0.022 | 0.843 | 0.113 | 0.310 | 0.010 | 0.923 | 0.025 | 0.834 | 0.091 | 0.478 |
| FC -> BI | -0.167 | 0.445 | -0.122 | 0.575 | -0.293 | 0.176 | -0.141 | 0.511 | -0.276 | 0.199 | 0.044 | 0.660 | -0.126 | 0.169 | 0.025 | 0.770 | -0.109 | 0.196 | -0.171 | 0.080 |
| FC -> Use | 0.127 | 0.130 | -0.031 | 0.779 | **0.218** | **0.014** | 0.097 | 0.226 | 0.164 | 0.080 | -0.157 | 0.103 | 0.092 | 0.148 | -0.029 | 0.574 | 0.038 | 0.585 | **0.249** | **0.014** |
| PE -> BI | **-0.331** | **0.021** | **-0.537** | **0.001** | **-0.330** | **0.022** | -0.218 | 0.107 | -0.229 | 0.126 | -0.206 | 0.086 | 0.002 | 0.985 | 0.114 | 0.193 | 0.103 | 0.348 | 0.207 | 0.087 |
| SI -> BI | 0.221 | 0.028 | **0.318** | **0.009** | 0.177 | 0.083 | 0.137 | 0.138 | **0.213** | **0.040** | 0.096 | 0.319 | -0.045 | 0.567 | -0.084 | 0.217 | -0.009 | 0.905 | -0.141 | 0.165 |

Appendix 2: Test PLS-MGA (continue)

|  | N14 - N4 | | N14 - N5 | | N15 - N4 | | N15 - N5 | | N4 - N5 | |
| --- | --- | --- | --- | --- | --- | --- | --- | --- | --- | --- |
|  | Diff | P-val. | Diff | P-val. | Diff | P-val. | Diff | P-val. | Diff | P-val. |
| BI -> Use | -0.005 | 0.974 | -0,035 | 0,687 | 0,053 | 0,273 | 0,023 | 0,726 | -0,030 | 0,570 |
| EE -> BI | -0.012 | 0.887 | 0,003 | 0,997 | -0,103 | 0,289 | -0,088 | 0,447 | 0,015 | 0,886 |
| FC -> BI | -0.019 | 0.848 | -0,154 | 0,090 | 0,152 | 0,079 | 0,017 | 0,833 | -0,135 | 0,087 |
| FC -> Use | 0.128 | 0.170 | 0,195 | 0,064 | **-0,121** | **0,041** | -0,054 | 0,479 | 0,067 | 0,313 |
| PE -> BI | **0.320** | **0.008** | **0,308** | **0,022** | 0,112 | 0,206 | 0,101 | 0,361 | -0,011 | 0,918 |
| SI -> BI | -0.180 | 0.058 | -0,105 | 0,305 | -0,039 | 0,597 | 0,036 | 0,674 | 0,075 | 0,317 |
